# Two distinct forms of human BLT2: long-form and short-form BLT2

**DOI:** 10.3389/fcell.2023.1288373

**Published:** 2023-10-26

**Authors:** Jun-Dong Wei, Jae-Hong Kim

**Affiliations:** ^1^ Department of Basic Medical Science, Medical College, Taizhou University, Taizhou, China; ^2^ Division of Life Sciences, College of Life Sciences, Korea University, Seoul, Republic of Korea

**Keywords:** leukotriene B_4_, lipid mediator, G protein-coupled receptor, BLT2, N-terminal domain

## Abstract

BLT2 is a low-affinity leukotriene B_4_ receptor that plays an essential role in the pathogenesis of various inflammatory diseases, including asthma and cancer. BLT2 is minimally expressed in a normal internal environment but is overexpressed in a stress-induced inflammatory environment. Recent research indicated that human BLT2 has two distinct forms. Although their functions are likely to be different, very few studies investigated these differences. Therefore, this paper will discuss about the two distinct forms of human BLT2; the short-form of BLT2 and the long-form of BLT2.

## 1 Introduction

Leukotriene B_4_ (LTB_4_) is a potent lipid mediator that is generated from arachidonic acid (Peters-Golden and Henderson, 2007). LTB_4_ triggers its activities via membrane G protein-coupled receptors (GPCRs), high-affinity LTB_4_ receptor (BLT1) and low-affinity LTB_4_ receptor (BLT2) (Saeki and Yokomizo, 2017). Unlike BLT1, BLT2 is minimally expressed in a normal internal environment but overexpressed in a stress-induced inflammatory environment such as cancer and asthma (Hennig et al., 2008; Park et al., 2016; Ro et al., 2019; Jang et al., 2021). Recent reports demonstrated that the LTB_4_-BLT2 axis plays a critical role in numerous inflammatory diseases, including asthma, atherosclerosis, and cancer (Sanchez-Galan et al., 2009; Ryu et al., 2010; Cho et al., 2013; Park et al., 2018; Kwon and Kim, 2019). Previously, we also observed that BLT2 induces ligand-dependent reactive oxygen species (ROS) generation (Choi et al., 2008; Cho et al., 2010a; Kim et al., 2010a). Furthermore, BLT2 overexpression was shown to be associated with an increase in LTB_4_-induced chemotactic responses through ROS-dependent manner (Wei et al., 2011). Human BLT2 (hBLT2) has shown to exist into two distinct forms and they may have some differences in functions (Kamohara et al., 2000; Tryselius et al., 2000; Wang et al., 2000; Yokomizo et al., 2000). In this review, we summarize a potential functional differences between human short-form of BLT2 (hSFBLT2) and human long-form of BLT2 (hLFBLT2), followed by a discussion on the structural and functional characteristics of hSFBLT2 and hLFBLT2.

## 2 Human short-form of BLT2

The hSFBLT2 consists of 358 amino acids (NM_019839.5) and was independently identified by three groups in 2000 (Kamohara et al., 2000; Wang et al., 2000; Yokomizo et al., 2000). hSFBLT2 shares 45.2% amino acid similarity with human BLT1 (hBLT1) (Table 1). The expression of hSFBLT2 was detected in various tissues, including the spleen, epithelial cells of the intestine, epidermal keratinocytes, and lungs (Yokomizo et al., 2000). Membrane preparations of CHO cells expressing hSFBLT2 showed binding to LTB_4_ with Kd values of 23 nM and could also be activated by various ligands, such as 12(*S*)-hydroxy-5(Z), 8(Z), 10(E), 14(Z)-eicosatetraenoic acid [12(*S*)-HETE], 12(*S*)-hydroperoxy- eicosatetraenoic acid [12(*S*)-HPETE], and 15(*S*)-hydroxyeicosatetraenoic acid [15(*S*)-HETE] at micromolar concentrations. In addition, 12(*S*)-hydroxyheptadeca-5(Z), 8(E), and 10(E)-trienoic acid (12-HHT) was identified as an effective agonist of hSFBLT2 in lipid fractions from the rat small intestine (Okuno et al., 2008). Using CHO cells membrane preparations overexpressing hSFBLT2, 12-HHT exhibited a high-affinity ligand (IC_50_ = 2.8 nM) for hSFBLT2 compared to LTB_4_ (IC_50_ = 25 nM) (Okuno et al., 2008). A recent study showed that 12-HHT activates Gi- and Gq-type G proteins, inducing hSFBLT2-mediated chemotaxis of mast cells (Okuno et al., 2008). Overexpression of hSFBLT2 activated with LTB_4_ leads to inhibition of cAMP production and elevation of intracellular calcium mobilization in CHO, COS7 cells. Pretreatment with pertussis toxin (PTX, an inhibitor of the Gαi family) eliminated these responses, indicating the coupling of hSFBLT2 with Gαi-type proteins. Furthermore, hSFBLT2 is also associated with LTB_4_-mediated chemotaxis via Gαi-type G-proteins (Iizuka et al., 2005). Recent studies showed the physiological roles of hSFBLT2, which included small intestinal barrier function and skin wound healing (Ishii et al., 2016; Storniolo et al., 2021). From the previous studies, we speculate that hSFBLT2 protects skin cells by participating in cell migration (Leguina-Ruzzi and Valderas, 2017).

**TABLE 1:** Characteristics of human long and short-forms of BLT2.

		hLFBLT2	References	hSFBLT2	References
GenBank accession no.		NM_019839.1		NM_019839.5	
Species (amino acid numbers)		Human (389)	Tryselius et al. (2000)	Human (358)	Kamohara et al. (2000), Wang et al. (2000), Yokomizo et al. (2000)
Ligand		12-HHT > LTB_4_ > 12(*S*)-HETE	Kim et al. (2020)	12-HHT ≫ LTB_4_ > 12(*S*)-HETE	Okuno et al. (2008)
Expression (human)		Liver >> pancreas, heart, lung, brain, kidney, placenta, and spleen	Tryselius et al. (2000)	Intestine, skin, spleen > endothelial cells, ovaries, liver, > ubiquitous	Yokomizo et al. (2000)
Coupled G-protein		Gi-type, Gq-type (in CHO-K1 cells)	Hennig et al. (2008) Wei et al. (2011)	Gi-type, Gq-type, Gz-type (in CHO-K1 cells)	Yokomizo et al. (2000) Okuno et al. (2008)
Function		Promotes various inflammatory diseases such as asthma and cancer	Cho et al. (2010a) Kim et al. (2010b) Kim et al. (2014a)	Barrier function and wound healing	Ishii et al. (2016) Leguina-Ruzzi and Valderas (2017)
Cell proliferation		++	Kim et al. (2014b)	+	Storniolo et al. (2021)
Chemotaxis	LTB_4_	+++	Wei et al. (2011) Kim et al. (2014b)	++	Yokomizo et al. (2000)
	12(*S*)-HETE	+	Sun et al. (2009)	+	Okuno et al. (2008)
	12-HHT	++	Kim et al. (2020)	+++	Okuno et al. (2008)
Wound healing		++	Sun et al. (2009)	++	Okuno et al. (2008)
ROS generation		+++	Wei et al. (2011) Kim et al. (2014a) Kim et al. (2014b)	+	Choi et al. (2008) Kim et al. (2014b)
Th2 cytokine production		++	Cho et al. (2010a) Kim et al. (2014b)	-	Kim et al. (2014b)

-, no effect; +, Weak; ++, Medium; +++, Strong; 12-HHT, 12(S)-hydroxyheptadeca-5(Z), 8(E), and 10(E)-trienoic acid; LTB_4_, Leukotriene B_4_; 12(*S*)-HETE, 12(*S*)-hydroxy-5(Z), 8(Z), 10(E), 14(Z)-eicosatetraenoic acid; ROS, reactive oxygen species; Th2, T helper 2.

## 3 Human long-form of BLT2

**FIGURE 1:**
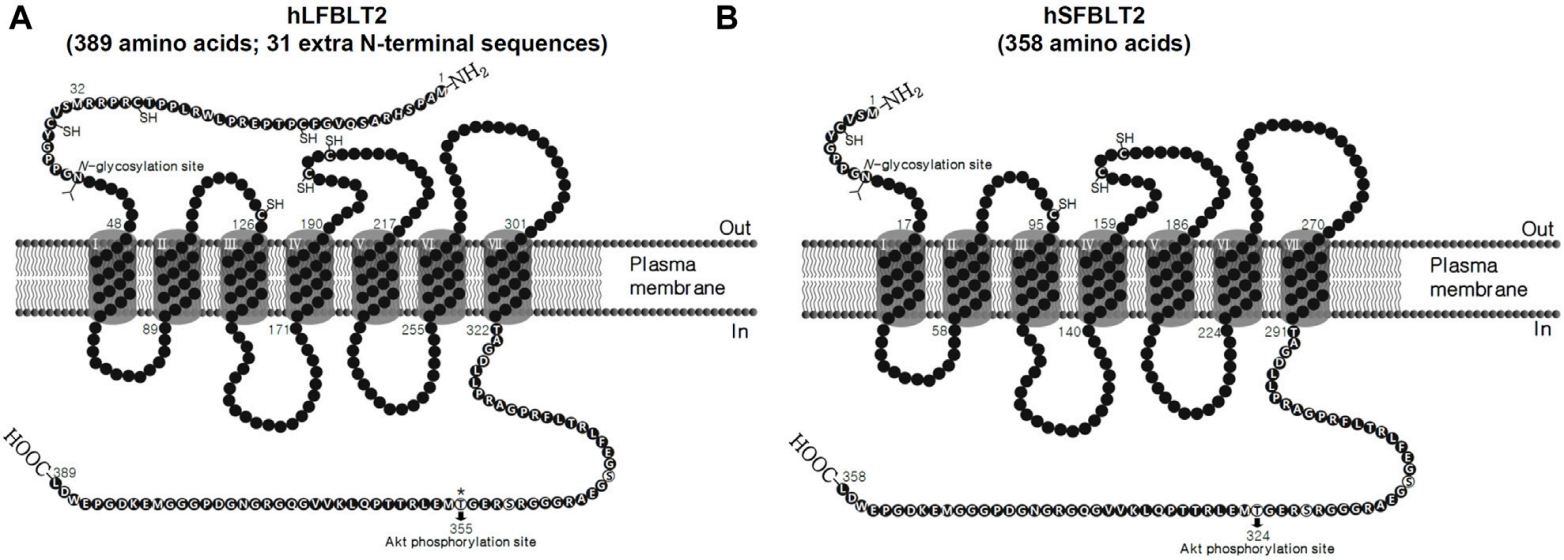
Comparison of N-terminal amino acid sequences between human long and short-forms of BLT2. **(A, B)** Topology model of human long-form of BLT2 and human short-form of BLT2 and the extra 31 amino acid residues in N-terminal of human long-form of BLT2.

The hLFBLT2 consists of 389 amino acids (NM_019839.1) and was first identified in 2000 (Tryselius et al., 2000). hLFBLT2 has 31 more amino acid residues at the N-terminal than the hSFBLT2 (Figure 1) and shares 39% amino acid with hBLT1 (Table 1). We found that LTB_4_ activated hLFBLT2, resulting in the activation of Gi-type G proteins (Kim et al., 2010b). hLFBLT2 also contributed to LTB_4_-dependent chemotaxis by stimulating Gαi-type G-proteins. Recent studies showed that hLFBLT2 was involved mainly in various inflammation diseases, such as cancer and asthma (Cho et al., 2010a; Kim et al., 2014a). Overexpression of hLFBLT2 markedly increased Nox1 mRNA levels, enhanced IκB-α phosphorylation, and the degradation of IκB-α in response to transforming growth factor (TGF)-β in mammary epithelial cells (Kim et al., 2014a). In addition, hLFBLT2 overexpression markedly enhanced TGF-β-induced epithelial-mesenchymal transition (EMT) in MCF-10A cell invasiveness (Kim et al., 2014a). Additionally, overexpression of hLFBLT2 leads to the increase of Bcl-2 levels and the decrease of Bax/Bad levels in detached PWR-1E cells, which rendered them resistant to anoikis, implicating hLFBLT2 as an essential determinant of resistance to anoikis in prostate cancer cells (Lee and Kim, 2013). We found that hLFBLT2 overexpression increased interleukin (IL)-8 transcript and protein levels and vascular endothelial growth factor (VEGF) cytokines in human mast cells (HMC-1) (data not shown). Additionally, activation of BLT2 enhanced IL-4 and IL-13 transcript and protein levels in bone marrow-derived mast cells (BMMCs) overexpressing hLFBLT2 (Cho et al., 2010a). We also found that, compared with hSFBLT2 overexpression, hLFBLT2 overexpression significantly enhanced LTB_4_-mediated chemotaxis in CHO-K1 cells (Kim et al., 2014b). Moreover, overexpression of hBLT2 in BMMCs, only BMMCs overexpressing hLFBLT2, rather than hSFBLT2, showed that LTB_4_ significant increased IL-13 transcript level (Cho et al., 2010a; Kim et al., 2014b). Taken together, hLFBLT2 expression levels are likely to be increased in inflammatory conditions, such as cancer and asthma, and may act as an inflammatory inducer to mediate inflammatory responses induced by LTB_4_.

## 4 Common features of hSFBLT2 and hLFBLT2

In general, both hSFBLT2 and hLFBLT2 are transcription products of the same BLT2 gene located on human chromosome14 at 14q11.2-q12. hLFBLT2 has 31 additional amino acids in the N-terminus. Both of them have helix 8, which acts as a pharmacological chaperone, promoting the surface expression of hBLT2 through passing the quality control in the endoplasmic reticulum (ER) (Yasuda et al., 2009). Recently, we tested various ligand efficacies for hLFBLT2, including LTB_4_, 12(*S*)-HETE, 12-HHT, and CAY10583 (a synthesis agonist for BLT2) using molecular modeling and demonstrated that, two amino acids, Tyr271 (equivalent to Tyr240 of hSFBLT2) and Asn275 (equivalent to Asn244 of hSFBLT2) in TM6 contribute to the activation of hLFBLT2 (Kim et al., 2020). Furthermore, recent study demonstrated the nuclear magnetic resonance (NMR) structure of 12-HHT in its hBLT2 bound-state (Giusti et al., 2020). In the docking simulation model, two amino acids, Ser174 of hSFBLT2 (equivalent to Ser205 of hLFBLT2) and Arg270 of hSFBLT2 (equivalent to Arg301 of hLFBLT2), were identified as the necessary residues to form hydrogen bonds in the interaction of hBLT2 and 12-HHT. Moreover, hSFBLT2 and hLFBLT2 have one N-glycosylation site, at Asn10 in hSFBLT2 and Asn41 in hLFBLT2. The loss of this N-glycosylation site leads to the accumulation of hBLT2 in the ER, implying that this modification is the necessity for the formation of hBLT2 in the ER (Yasuda et al., 2009). Additionally, there is a residue Thr355 in hLFBLT2 (equivalent to Thr324 in hSFBLT2) in the cytoplasmic region of hBLT2, which is phosphorylated by AKT and contributes to BLT2-mediated chemotactic responses (Wei et al., 2011). We also showed that RanBPM, a scaffolding protein, interacted with the C-terminal domain of hLFBLT2 to inhibit internalization of LTB_4_-triggered hLFBLT2 (Wei et al., 2013). Moreover, the C-terminal domain of hBLT2 is essential for its transport from the Golgi apparatus to the lateral membrane in a membrane trafficking protein Lin-7 homolog C (LIN7C)-dependent manner, contributing to barrier formation in epithelial cells (Hara et al., 2021).

## 5 N-terminal domain of hLFBLT2

The N-terminal domain of GPCR plays a pivotal role in ligand recognition, receptor activation, and receptor structure (Couvineau and Laburthe, 2012). In addition, the disulfide bond links the N-terminus and the extracellular loop (ECL) of the GPCR strongly influences ligand binding as well as cellular responses (Szpakowska et al., 2014). Indeed, the cysteines mutation at the N-terminus and ECL attenuated ligand-receptor binding and ligand-induced signaling transduction in the chemokine receptor (Limatola et al., 2005). Additionally, the constraint exerted by the disulfide bond may form the ligand binding pocket and/or be important in the correct position for ligand interactions with the receptor (Szpakowska et al., 2012; Sonawani et al., 2022). The N-terminal domain of hLFBLT2 contains three cysteine residues, one cysteine residue in ECL1, and two cysteine residues in ECL2 (Figure 1). It is possible to form disulfide bridges at the N-terminal and ECL of hLFBLT2, which may play an essential role in the formation of the ligand-binding pocket and the activation of receptors. Recently, we found that 196 amino acid in the ECL2 of hLFBLT2 was mutated from Asp to Gly, increased ligand binding affinity and cell motility under low-dose stimulation of its ligands (Jang et al., 2017). Using the online software ProtScale to predict the hydropathicity of the N-terminal domain of hLFBLT2, we found that compared to hSFBLT2, the N-terminal domain of hLFBLT2 has a hydrophobic region. Recent report indicated that the hydrophobic region at the N-terminus of the receptor increases receptor trafficking via the early secretory pathway, without a significant influence on overall receptor biosynthesis in the case of the rat corticotropin-releasing factor receptor type 1 (CRF1R) (Rutz et al., 2006). The hydrophobic region at the N-terminal domain of hLFBLT2 may promote transport through the early secretory pathway, thus increasing the expression of receptor under inflammatory conditions to better adapt to increases in pro-inflammatory factors, such as LTB_4_. Moreover, in various experiments with BLT2 ligand [LTB_4_, 12(*S*)-HETE or 12-HHT]-induced ROS generation and cell chemotaxis, we also found that LTB_4_-mediated chemotaxis had a significant effect in CHO-K1 cells overexpressing hLFBLT2 compared to hSFBLT2, indicating that the N-terminal of hLFBLT2 has more selective to LTB_4_ in ligand-mediated ROS generation and chemotaxis (Kim et al., 2014b). Recent studies suggested that overexpression of hLFBLT2 play a critical role in promoting tumor progression such as proliferation, survival, migration, and metastasis (Lee and Kim, 2013; Kim et al., 2014a; Wei et al., 2017). These findings indicate that LTB_4_ triggers its physiological and pathophysiological functions *in vivo* through hLFBLT2. However, further investigation is required to ascertain whether disparities exist in the regulation of intracellular signal transduction molecules downstream of hBLT2. Specifically, it is necessary to determine if variations in ligand affinity, cAMP signaling, or calcium flux exist between hLFBLT2 and hSFBLT2 under identical conditions, and whether these disparities are influenced by cell type or disease induction. This inquiry aids in attaining a more comprehensive comprehension of the functional distinctions between the two forms of BLT2.

## 6 hLFBLT2 is exclusive to humans

Recent research has shown that BLT2 can be expressed in other species except humans including mouse, rats, and zebrafish, but hLFBLT2 seems to be specifically expressed in humans. It has also been found in various types of cells, such as mast cells, dendritic cells, epithelial cells, and cancer cells (Table 2). Unlike human, only one form of mouse BLT2 (mBLT2) has been reported in mice, which share 92% sequence homology with hSFBLT2 (Iizuka et al., 2005). mBLT2 was mainly expressed in epithelial cells of skin, the small intestine, colon, and cornea (Iizuka et al., 2010; Liu et al., 2014; Iwamoto et al., 2017). The expression of mBLT2 was detected in epithelial and cryptic cells of the mouse intestine by *in situ* hybridization analysis (Iizuka et al., 2010). In mouse dextran sodium sulfate (DSS)-induced colitis model, BLT2-knockout (KO) mice exhibited severe body weight loss and intestinal inflammation compared to wild-type (WT) mice. Overexpression with mBLT2 in Madin-Darby canine kidney II (MDCK II) cells increased trans-epithelial resistance, implying increased barrier activity (Iizuka et al., 2010). In a murine asthma model, we found that mBLT2 expression was increased in the lungs after ovalbumin (OVA) challenge. In addition, treatment with LY255283 (a BLT2 antagonist) inhibited airway inflammation, airway hyperresponsiveness (AHR), ROS generation, and the expression of NF-κB (Cho et al., 2010b). In mouse pneumolysin (PLY, a pneumococcal toxin)-induced acute lung injury model, BLT2-KO mice showed higher sensitivity to intratracheal injection of PLY and died due to enhanced vascular permeability and bronchoconstriction (Shigematsu et al., 2016). In an OVA-induced allergic airway mouse disease model, BLT2-KO mice also exhibited severe eosinophilic lung inflammation due to increased IL-13 production of BLT2-KO CD4^+^ T-cells (Matsunaga et al., 2013). Recently, we found that BLT2-KO suppresses KrasG12D-driven lung inflammation, IL-6 production, and tumor formation in a KrasG12D/BLT2-KO double-mutant mouse model compared to KrasG12D mice (Jang et al., 2021). In the mouse model of allergic asthma, we found that mBLT2 was upregulated in mast cells stimulated by allergen and mediated Th2 cytokines production such as IL-4 and IL-13 (Cho et al., 2010a). Compared with hBLT2, the biological function of mBLT2 may depend on its ligand stimulation *in vivo* to a certain extent. High level of 12-HHT was detected in the epidermal keratinocytes of mouse skin, and mBLT2 was involved in 12-HHT-mediated cell migration, which was crucial for wound healing of skin cells (Liu et al., 2014). However, in inflammatory state, with the increase of LTB_4_ level, mBLT2 can participate in LTB_4_-induced inflammatory response, which can play a key role in the pathogenesis of these inflammatory diseases, such as cancer and asthma.

**TABLE 2:** Cell-types and biological function of BLT2.

Cell type	Function	References
Bone marrow derived mast cell	Contributes to allergic airway inflammation	Kwon and Kim (2019) Cho et al. (2010a) Lee et al. (2017) Ro et al. (2018)
Dendritic cell	Induces cell chemotaxis	Shin et al. (2006)
Mouse macrophage	Contributes to LPS-induced IL-6 production	Lee et al. (2015)
Human natural killer (NK) cells	Induces cell migration	Wang et al. (2015)
Chronic myeloid leukemia	Promotes leukemogenesis by maintain cell growth and survival	Xiao et al. (2016)
Keratinocyte	Promotes skin wound healing and re-epithelialization	Iwamoto et al. (2017) Liu et al. (2014)
Bronchial epithelial cells	Enhances cell proliferation and migration	Liu et al. (2018)
Intestinal epithelial cells	Increases cell migration and accelerats wound healing	Ishii et al. (2016) Matsumoto et al. (2020)
Colon cryptic cells	Enhances barrier function in epithelial cells of the colon	Iizuka et al. (2010)
Human cancer cells	Involves in cancer progression such as proliferation, invasion, and metastasis	Hennig et al. (2008) Jang et al. (2021) Park et al. (2018) Seo et al. (2011); Lee et al. (2012); Park et al. (2019)

Rat BLT2 (rBLT2) was also reported to exist as only one form. Primary rat myoblasts expressed rBLT2, and treatment with LY255283 did not reduce the acceleration of LTB_4_-induced myoblast proliferation, suggesting that rBLT2 did not affect LTB_4_-induced rat myoblast proliferation and differentiation (Sun et al., 2009). Expression of the rBLT2 mRNA was identified in the thymus, synovium, spleen, and peripheral blood mononuclear cells (PBMCs), and mediated the effect of LTB_4_ in the progression of synovitis. Increases in basal levels of IL-4 and IL-13 were detected in lung tissue overexpressing transgenic (TG) rBLT2, suggesting that rBLT2 may be responsible for the enhancement of AHR, as assessed by lung resistance (R_L_), contributing to the OVA-induced allergic response (Cho et al., 2010a). Recent studies showed rBLT2 mRNA expression in the synovium of collagen-induced arthritis (CIA) model rats, indicating that rBLT2 may play an essential role in different stages of inflammatory arthritis (Han et al., 2007).

Two BLT2-type receptors, zebrafish BLT2a (zBLT2a) and zebrafish BLT2b (zBLT2b) were identified in 2015 (Okuno et al., 2015). The amino acid sequences of zebrafish share 29%–34% with hSFBLT2. In zebrafish, the zBLT2a genes are located on chromosome 7, and the zBLT2b gene is located on chromosome 2. Cells expressing zBLT2a and zBLT2b exhibited LTB_4_- and 12-HHT -induced intracellular calcium mobilization, inhibition of cAMP formation, [^35^S]GTPγS binding, and TGFα-shedding activity. The intracellular free (Ca^2+^) showed that 12-HHT activated zBLT2a and zBLT2b at lower doses than LTB_4_ (Okuno et al., 2015). However, the biological function of zBLT2 has not been reported.

## 7 Conclusion

In general, hSFBLT2 and hLFBLT2 have similar characteristics and are transcription products of the same BLT2 gene located on human chromosome 14 at 14q11.2-q12. At present, the mechanism of transcriptional regulation of these two forms of BLT2 is still unclear, and there is no antibody available to effectively distinguish the two substances *in vivo*. However, in terms of function, both are coupled Gi-type proteins that can be activated by LTB_4_ and contribute to similar intracellular signaling, such as chemotaxis and wound healing. Under normal conditions, activation of hSFBLT2 participates in the movement of various cells, including wound healing in the skin and barrier function in the small intestine, which is related to the recovery of skin cells. In contrast, hLFBLT2 is induced in the inflammatory environment, and is believed to further aggravate inflammation and thus, promote inflammatory diseases. LTB_4_ exerts its biological functions *in vivo*, perhaps through hLFBLT2, in inflammatory conditions. To gain a deeper comprehension of the involvement of BLT2 in human inflammatory diseases, it would be advantageous to construct humanized transgenic mouse models that express hLFBLT2 or hSFBLT2, and subsequently assess their phenotype. Therefore, it is desirable to distinguish the two different types of hBLT2 and develop drugs that specifically inhibit hLFBLT2 to treat human inflammatory diseases.
